# Central tendency effects in time interval reproduction in autism

**DOI:** 10.1038/srep28570

**Published:** 2016-06-28

**Authors:** Themelis Karaminis, Guido Marco Cicchini, Louise Neil, Giulia Cappagli, David Aagten-Murphy, David Burr, Elizabeth Pellicano

**Affiliations:** 1 Centre for Research in Autism and Education (CRAE), Department of Psychology and Human Development, UCL Institute of Education, University College London, London, WC1H 0NU, UK; 2 School of Psychology, Plymouth University, Plymouth, PL4 8AA, UK; 3 Institute of Neuroscience, National Research Council (CNR), Pisa, 56124, Italy; 4 Istituto Italiano di Tecnologia, Genova, 16163, Italy; 5 Department of Psychology, Ludwig-Maximilians-Universität München, Münich, 80802, Germany; 6 School of Psychology, University of Western Australia, Crawley, Perth, Western Australia, 6009, Australia

## Abstract

Central tendency, the tendency of judgements of quantities (lengths, durations etc.) to gravitate towards their mean, is one of the most robust perceptual effects. A Bayesian account has recently suggested that central tendency reflects the integration of noisy sensory estimates with prior knowledge representations of a mean stimulus, serving to improve performance. The process is flexible, so prior knowledge is weighted more heavily when sensory estimates are imprecise, requiring more integration to reduce noise. In this study we measure central tendency in autism to evaluate a recent theoretical hypothesis suggesting that autistic perception relies less on prior knowledge representations than typical perception. If true, autistic children should show reduced central tendency than theoretically predicted from their temporal resolution. We tested autistic and age- and ability-matched typical children in two child-friendly tasks: (1) a time interval reproduction task, measuring central tendency in the temporal domain; and (2) a time discrimination task, assessing temporal resolution. Central tendency reduced with age in typical development, while temporal resolution improved. Autistic children performed far worse in temporal discrimination than the matched controls. Computational simulations suggested that central tendency was much less in autistic children than predicted by theoretical modelling, given their poor temporal resolution.

The ability to estimate time is important in many everyday situations, from crossing a busy street to turn-taking in conversations and social interactions. Timing abilities are also involved in the development of other cognitive skills. For example, it has been proposed that time-processing difficulties underlie the atypical organisation of the phonological system in children with language impairments, which in turn affects other aspects of language and reading development in these children^1^. Anecdotal reports suggest that autistic individuals also have a particularly poor sense of time, presenting difficulties in gauging how much time has passed^2^,^3^. However, the nature and the extent and the implications of timing difficulties on the development of the autistic child are less well understood^4^.

Like other judgements of quantities (e.g., length, number, colour, etc.), time duration estimates present the characteristic tendency to gravitate towards their mean value, often referred to as central tendency^5^,^6^. In the temporal domain, relatively short or relatively long time intervals will be over- or under-estimated, while duration estimates of the same time intervals will be shorter or longer depending on whether they are presented in the context of short or long intervals (context-dependency^7^).

A Bayesian account has recently been put forward to explain central tendency effects in time interval reproduction^8^,^9^. According to this account, the magnitude of central tendency reflects the extent to which noisy sensory signals (‘likelihoods’ of sensory observations) are integrated with internal representations of a mean value (‘prior knowledge’ about temporal statistics) to generate final (‘posterior’) perceptual judgements. The more noisy and ambiguous the sensory estimates the more final perceptual judgements rely on prior knowledge representations, representing a trade-off between accuracy and reliability (some accuracy sacrificed in favour of improvements in reliability). Priors therefore improve the efficiency of computations by reducing overall noise or error.

This Bayesian account of central tendency explained the patterns of performance of participants with different levels of musical training in a time interval reproduction task (measuring central tendency) and a time bisection task (measuring temporal resolution) presented across two modalities, auditory or visual^8^,^10^. For example, in the visual modality (time intervals presented using flashes), non-musician adults and string musicians showed lower temporal resolution and increased central tendency compared to percussionists, who reproduced temporal intervals veridically. By contrast, in the auditory modality (time intervals presented using ‘beep’ sounds), all three groups presented very high temporal resolution and negligible central tendency effects, i.e., they reproduced intervals either veridically or with a systematic bias. Computational modelling of the combined results (two tasks, three groups of participants, two modalities) suggested that prior knowledge and sensory signals were integrated flexibly, i.e. with differential weighting, across groups and across modalities in a way that achieved the minimisation of relative error in all cases (i.e. it was optimal, according to the ideal observer model).

Two outstanding questions are how central tendency effects such as those studied in the study of Cicchini *et al*.^8^ manifest across development and how these effects differ if individual variability in cognitive and perceptual development is taken into account. With regards to the first question, no existing studies (to our knowledge) have assessed the development of central tendency per se in time interval reproduction. However, developmental studies have shown that children perform better with age in time interval reproduction tasks, presenting mainly improvements in precision rather than accuracy^11^,^12^. Studies have also shown that temporal resolution abilities are gradually refined in childhood, presenting marked changes between the ages of 5 and 8 years^13^,^14^,^15^.

A recent study by Sciutti *et al*.^7^ focused on central tendency and context dependency effects in typical development in the spatial domain. These authors administered a child-appropriate spatial interval reproduction/spatial interval discrimination paradigm to 7 to 14 year-old children and adults. Both child and adult participants presented central tendency and context dependency effects in the spatial interval reproduction task and the magnitude of these did not change with age. Computational modelling of the cross-sectional data suggested that children weighted prior knowledge about the mean with sensory signals in a constant manner across development and similarly to adults. Given that children presented marked developmental improvements in their spatial resolution (spatial interval discrimination task), Sciutti *et al*.^7^ argued that the Bayesian “use of prior knowledge” strategy is useful for restraining sensory noise across development.

For the second question, how central tendency effects might differ because of individual variability in cognitive and perceptual development, it is particularly relevant to examine these effects in children diagnosed with autism. Autism is a highly heterogeneous neurodevelopmental condition, which is perhaps better known for its striking effects on social interaction and communication. Yet, autism is also characterised by atypicalities in sensation and perception^16^,^17^,^18^. Anecdotal reports suggest that, autistic individuals have difficulties with time, particularly in gauging how much time has passed^2^,^3^. However, studies that have experimentally tested time processing in autistic individuals have produced mixed and inconclusive results^4^. For example, a study by Maister and Plaisted-Grant^19^ showed that while children aged 8 to 13 years were less accurate and more variable than age- and ability-matched typical children in the reproduction of short (<2 sec) and long (>45 sec) stimuli presented in the visual modality, no difficulties were found in reproducing durations 4–30 sec. Difficulties with temporal interval reproduction in autism have also been reported in children/adolescents^20^ and adults^21^, however other studies^22^ have found no such difficulties. Furthermore, with regards to temporal resolution, some studies^23^,^24^ found reduced resolution in children and adolescents with autism, while others^22^,^25^ found no such difficulties.

The explicit examination of the role of prior information in perception is important to the theoretical understanding of autism. A recent account of autistic perception by Pellicano and Burr^26^ (see also related accounts^27^,^28^,^29^,^30^,^31^) has proposed that less precise or attenuated priors, within a model of Bayesian perceptual inference, might be responsible for the unique perceptual experiences of autistic people. Attenuated priors should lead to a tendency to perceive the world in a more ‘accurate’ way, in the sense that sensory signals are modulated to a lesser extent by prior experience, resulting in less systematic biases in perception (note that increased accuracy does not necessarily imply overall ‘better’ perception, see Teufel *et al*.^29^ for a discussion). A direct implication is that autistic children should present reduced central tendency effects in a time interval reproduction task, than those predicted by the Bayesian model of central tendency given their temporal resolution.

In the current study, we evaluated this prediction with a developmentally appropriate and engaging version of the time interval reproduction task used by Cicchini *et al*.^8^, which measured central tendency and error of accuracy and reliability in time reproduction; together with a time discrimination task, which assessed temporal resolution (Fig. 1). We first established the typical developmental trajectory for central tendency effects in time interval reproduction by administering our paradigm to typically developing children in the age range 6 to 14 years old and adults (similar to the study of Sciutti *et al*.^7^ on spatial interval reproduction). Next, we examined central tendency effects in a group of autistic children between 6 to 14 years old and compared their performance to a subset of the typically developing children who took part in the study, of similar age and language and reasoning abilities. Finally, we employed computational modelling techniques used in the previous studies^7^,^8^ to assess the relative weighting of prior knowledge and sensory input in developmental groups and children with and without autism and the extent to which the integration of the two sources of information in different groups served optimal computations.

## Results

First, we present results for the performance of the different groups in the temporal interval reproduction task. Next, we examine the performance of the different groups in the time discrimination task. Finally, we focus on the computational modelling of the combined data from the two tasks.

### Time interval reproduction

#### Raw data

Figure 2 presents the uncorrected reproduced times (raw data) in the two sessions of the interval reproduction task for different participant groups, along with the lines fitted to the pooled data from each session. Panels a to e focus on typically developing children and adults, while panels F and G show raw data for the age-and ability matched groups of autistic and typical children.

Visual inspection suggests that all participants showed central tendency effects, as the slope of the fitted lines was less steep than the equality line (all *ps* < 0.05). All groups also tended to underestimate stimuli intervals, that is the data lying below the equality line, but this did not affect the regressions (systematic errors are separable from central tendency, see Supplementary Method for a discussion). Context-dependency effects (see Supplementary Method), evidenced by longer reproduction times in the long than in the short interval condition for the stimuli that were common in the two sessions, were found in the combined data from the groups of typically developing children and adults, *t*(77) = 3.43, *p* = 0.001, *d* = 0.39. Unexpectedly, however, these effects were not consistent across different groups. The difference between estimates for the common stimuli in the long and the short interval condition was significantly greater than zero only for the younger groups of typical children [6–7 year-olds: *t*(11) = 2.16, *p* = 0.05, *d* = 0.63; 8–9 year olds: *t*(18) = 4.00, *p* = 0.001, *d* = 0.92] and autistic children [*t*(22) = 2.52, *p* = 0.02, *d* = 0.53], but not for the older groups of typical children [10–11 year-olds: *t*(17) = −0.19, *p* = 0.85, *d* = −0.04; 12–14 year olds: *t*(14) = −0.51, *p* = 0.63, *d* = −0.13], adults [*t*(13) = 2.00, *p* = 0.07, *d* = 0.53] and the typical comparison children [*t*(22) = 0.92, *t* = 0.37, *d* = 0.19]. Context dependency effects in our data were therefore not as strong as in the developmental study of Sciutti *et al*.^7^ in spatial interval reproduction.

#### Regression Indices

Figure 3 shows regression indices (the difference between the slope of a fitted line and the equity line; see Analysis of empirical data and Supplementary Method), a measure of the magnitude of central tendency (see Analysis of empirical data), for the short and long conditions of the temporal interval reproduction task (3a: typical children and adults; 3b: age- and ability matched autistic and typical children). Regression indices were high in all age groups and tended to decrease with age in typical development. This observation was confirmed by a mixed-design ANOVA on regression indices with age group (6–7 year-olds, 8–9 year-olds, 10–11 year olds, 12–14 year-olds, and adults) as a between-participants factor, and interval condition (short, long) as the within-participants factor. There was a significant main effect of age group, *F*(4, 73) = 10.46, *p* < 0.001, *η*^2^ = 0.36, but no significant effect of interval condition, *F*(1, 73) = 0.01, *p* = 0.94, *η*^2^ = 0.00, and no significant age group X interval condition interaction, *F*(4, 73) = 1.86, *p* = 0.12, *η*^2^ = 0.09.

Autistic children showed significantly higher levels of central tendency than the comparison children (average across sessions for autistic children: *M* = 0.61; *SD* = 0.21; average for typical comparison children: *M* = 0.45; *SD* = 0.18). A mixed-design ANOVA on the regression indices shown in Fig. 3b yielded a significant main effect of group, *F*(1, 44) = 7.86, *p* = 0.01, *η*^2^ = 0.15, a significant effect of interval condition, *F*(1, 44) = 4.85, *p* = 0.03, *η*^2^ = 0.10, and no significant interaction between the two factors, *F*(1, 44) = 0.37, *p* = 0.55, *η*^2^ = 0.01. The regression indices of autistic children were roughly comparable to those presented by the younger typical group of 8–9 year-olds.

We also examined the build-up of regression across the session by performing a split-half analysis in which we compared regression indices estimated from data from the first and the second halves of the sessions (Supplementary Figure 1). The data of typically developing children and adults suggested that regression indices increased from the first to the second half, *F*(1, 73) = 6.46, *p* = 0.01, *η*^2^ = 0.08, suggesting the learning or the progressive use of a prior during the progression of the session. The data of autistic and comparison children also presented evidence of learning of a prior, *F*(1, 44) = 7.42, *p* = 0.01, *η*^2^ = 0.17. The differences in regression indices between the two groups were comparable, *F*(1, 44) = 1.48, *p* = 0.23, *η*^2^ = 0.15, while the group x session half interaction was not statistically significant, *F*(1, 44) = 1.49, *p* = 0.23, *η*^2^ = 0.04, suggesting that the learning of a prior across a session was similar in the autistic and the comparison children.

#### Reproduction Error

Figure 4 plots the total average reproduction error (combined error of accuracy and reliability, see Supplementary Method) in the short and the long interval conditions of the reproduction task. In typical development (subplot a), the two measurements improved with age, as expected. Error was slightly higher in the short interval condition, possibly because a smaller interval value is used for normalisation (see Measurements and analysis in Supplementary method available). A mixed-design ANOVA with total error as the dependent variable, age group as a between-participants factor and interval condition as a within-participants factor, showed a significant main effect of age group, *F*(4, 73) = 7.86, *p* < 0.001, *η*^2^ = 0.30, and interval condition, *F*(1, 73) = 4.95, *p* = 0.03, *η*^2^ = 0.05, and no significant age x interval condition interaction, *F*(4, 73) = 1.57, *p* = 0.19, *η*^2^ = 0.07.

Interestingly, the age- and ability-matched groups of autistic and typical children did not differ with respect to error. A mixed-design ANOVA, with group as a between-participants factor and interval condition as a within-participants factor, suggested that a trend for higher total error in autistic children was not significant, *F*(1, 44) = 3.63, *p* = 0.06, *η*^2^ = 0.08. The same analysis found no effects of interval condition, *F*(1, 44) = 7.35, *p* = 0.10, *η*^2^ = 0.14, and no significant interactions between group and interval condition, *F*(1, 43) = 0.52, *p* = 0.46, *η*^2^ = 0.01.

We also examined between group differences in the two orthogonal components of error (see Analysis of empirical data and Supplementary Method), the coefficient of variation (CV, error related to the reliability of responses) and BIAS (error related to accuracy). The empirical data for the two components of error are shown in Fig. 5 (circles: typical children and adults; squares: autistic and comparison children; lines: see Computational Modelling). Mixed-design ANOVAs, with age group as a between-participants factor and interval condition as a within-participants factor, yielded significant main effects of age group on both components [BIAS: *F*(4, 73) = 11.58, *p* < 0.001, *η*^2^ = 0.39; CV: *F*(4, 73) = 5.68, *p* < 0.001, *η*^2^ = 0.24] as well as on the ratio CV/BIAS, *F*(4, 73) = 3.44, *p* = 0.01, *η*^2^ = 0.16. Bonferroni post-hoc comparisons suggested that the rate CV/BIAS was lower in the 6–8 year-olds than adults (*p* = 0.01), however, all other pairwise comparisons were not significant. Overall, our findings suggested that typical children become more precise and more accurate with age in time interval reproduction. The modelling of these data (continuous lines) will be discussed in the Computational Modelling section.

The orange and light blue squares of Fig. 5 show respectively the data for the autistic participants and the matched typical children. Mixed-design ANOVAs with group as a between-participants factor and interval condition as a within-participants factor showed no significant effect of group on CV, *F*(1, 44) = 2.43, *p* = 0.13, *η*^2^ = 0.05, but a significant effect of group on BIAS, *F*(1, 44) = 7.08, *p* = 0.01, *η*^2^ = 0.14. There was, however, no significant main effect of group on the ratio CV/BIAS, *F*(1, 44) = 1.13, *p* = 0.22, *η*^2^ = 0.03. In summary, autistic children were less accurate than but equally precise as the typical comparison children in time interval reproduction, although this difference in accuracy did not translate to a significant between group difference in total error or the ratio CV/BIAS, our measure for evaluating potential differences between groups in terms of their strategy in the time interval reproduction task (e.g., more accurate but more variable vs. less accurate but less variable).

### Time discrimination task

Figure 6 plots the temporal resolution of the groups of typical children and adults and the two age- and ability matched groups, given by the Weber fractions obtained from the time discrimination task. Temporal resolution presented marked improvements with age in typical development and a one-way ANOVA showed a significant effect of age group on Weber Fraction, *F*(4, 73) = 4.31, *p* = 0.003. Autistic children obtained far higher Weber fractions (*M* = 0.36, *SD* = 0.21) than the typical comparison children (*M* = 0.22, *SD* = 0.13), *t*(44) = 2.68, *p* = 0.01, *d* = 0.80. The temporal resolution of autistic children was comparable to the youngest group of typical children.

### Summary of empirical results

The combined findings from the two tasks suggested that, in typical development, central tendency effects decrease with age, while temporal resolution improves. Central tendency effects are higher in autistic children compared to the typical comparison children, and autistic children performed worse in temporal discrimination. As the performance of autistic children was similar to that of younger children in both tasks, it is possible that their performance results from a general developmental delay.

Yet autistic children performed far worse than the typical comparison children in the time discrimination task, with comparable precision to the youngest group of children. Comparing the autistic group with the youngest group of typical children suggests that they have similar temporal discrimination, but less central tendency. This pattern could suggest atypicalities related to the use of central tendency strategies per se in autism (consistent with Pellicano and Burr^26^), rather than general delayed maturation of the time reproduction and discrimination abilities.

We examined this possibility using the computational modelling procedures of Cicchini *et al*.^8^ and Sciutti *et al*.^7^. Such procedures aimed to assess: i) the strength of prior knowledge representations for temporal statistics (the wider the normally distributed priors, the less strong the representations) that the different groups used in our paradigm, and ii) the extent to which these representations helped participants to achieve optimal performance in the temporal interval reproduction task.

### Computational modelling

#### Modelling performance in time interval reproduction under the assumption of different prior widths

The continuous lines of Fig. 7 plot model predictions of how regression indexes should vary with Weber fraction (time discrimination task), for four different Gaussian priors, with widths (i.e., 1 SD), ranging from 100 to 400 ms. The model predicted regression indices similar to the empirical data of different groups when using prior width values ranging from 400 ms to 200 ms for all groups of participants. This simulation also predicted a pattern of prior representations becoming broader with age in typical development. The data of autistic children (orange square) fell on the curve generated by the broadest prior (400 ms) used in the simulation. Furthermore, the prior width that captured the data of autistic children was considerably broader than the prior width predicted for the 6–7 year-olds, who presented similar levels of temporal resolution as autistic children. This finding also held when the two conditions, short and long, were analysed separately (Supplementary Figure 2). In other words, the model predicts much broader (weaker) priors for the autistic participants, consistent with the predictions of Pellicano and Burr^26^.

Figure 5 plots model predictions, together with data, for the two separate components of reproduction error in the interval reproduction task (see Supplementary Figure 3 for modelling results from the short and the long condition separately). Like the simulation of regression indices, the model predicted rates of CV and BIAS that were similar to the empirical data, for prior widths between 400 ms and 200 ms. However, the pattern of prior widths predicted for the autism group were broader than those predicted for the youngest group of children.

#### Assessing relative error

Figure 8 shows the predicted error landscape as a function of Weber fraction and prior width (see Supplementary Figure 4 for results from the short and the long condition). The developmental data showed a continuous improvement of optimality with age, manifested by the progression to colder areas of the error surface. Similar to Cicchini *et al*.^8^, the adult data lay closer to the minimal error areas of the surface, suggesting optimal computations. The estimates of prior widths for the different groups ranged from around 400 ms to 200 ms.

The autistic children (orange squares) were clear outliers on this plot, with far reduced efficiency compared with both their comparison group (light blue squares), and also the ability-matched 6-year-old group (red circles). The autistic children had far weaker (broader) priors than ideal for maximum efficiency, as predicted by Pellicano and Burr^26^.

## Discussion

Anecdotal reports suggest that autistic individuals have a particularly poor sense of time and present characteristic difficulties in keeping track of how much time has passed^2^,^3^. Some researchers have proposed that timing difficulties are a preeminent feature of autistic perception^32^, which manifests in the need of autistic people to schedule future events and their distress when plans change^3^, as well as in atypicalities in other cognitive domains and difficulties in social interaction^33^. Yet, empirical research on the timing abilities of autistic individuals has produced mixed results (studies showing limitations in time processing^23^,^24^ vs. studies showing no difficulties^22^,^25^). Given this inconclusive evidence, it is difficult to evaluate the nature and extent of timing difficulties in, as is to provide a rigorous account of how an atypical sense of time (if any) might relate to the broader autistic phenotype.

In the current study, we focused on a particular aspect of time estimation of autistic children, namely central tendency in time interval reproduction. This tendency of quantity judgements to gravitate towards their mean value^5^ has been recently modelled within the Bayesian framework of perceptual inference. Within this framework, the magnitude of central tendency reflects the flexible integration of noisy sensory estimates with internal representations for the mean value of stimuli to produce final judgements. In this study, we applied this Bayesian model of central tendency in time interval reproduction to characterise performance in typical development and in autism.

Our time interval reproduction data showed that in typically developing children central tendency effects occur at very young ages, and decrease with age. These results parallel those of Sciutti *et al*.^7^, who showed a similar reduction in regression between the ages of 7 and 10 years for a spatial interval reproduction task. Our findings therefore suggest that not only are young children able to extract statistical information for the recent history of sensory input; they do so to a greater extent than older children and adults.

At a first instance, this finding seems counterintuitive. However, there is considerable evidence that abilities for statistical learning are available very early in development, even in infants^34^,^35^ and newborns^36^. Such statistical learning abilities are thought to tap on mechanisms for experience-dependent plasticity, which are important for cognitive development, for example language acquisition^37^,^38^. Arguably, the pattern of higher regression in younger children in our data could reflect the greater reliance upon statistical learning at younger ages.

As regression decreased with age, temporal discrimination improved, agreeing with previous results^13^,^14^,^15^,^39^,^40^,^41^ and the study of Sciutti *et al*.^7^ on the spatial domain. The computational simulations combined developmental measures of central tendency and temporal resolution to quantify the strength of prior knowledge representations and how these compared to optimal computations. The simulations suggested that typically developing children tend to perform near optimally, with the younger children using stronger (narrower) priors, consistent with their poorer temporal resolution. The integration of noisy sensory estimates with internal representations for the mean value of stimuli is therefore flexible in childhood. As Sciutti *et al*.^7^ discuss central tendency serves to restrain the continuously decreasing sensory noise.

The data of the autistic group revealed two main facts: (1) autistic children do show regression, and indeed, at higher levels than the age- and ability matched typical children; (2) but at the same time, their temporal thresholds are about twice as high as those of the matched-typical group and very similar to the 6-year-olds. This pattern implies that the use of priors in autistic children does not increase enough to compensate for the lower precision and instead remains very similar to the typical comparison group.

The elevated levels of error of reliability and error of accuracy of autistic children in the time interval reproduction task are consistent with results of other studies using time reproduction tasks^22^,^23^. By contrast, the decreased temporal resolution of autistic children (time discrimination task) is congruent with some studies^24^ but not with others^22^,^25^,^42^. More research is warranted in order to unify this range of inconclusive findings on temporal resolution of autistic children. The role of methodological differences - for example the range of time intervals tested, modality (auditory in studies by Mostofsky *et al*.^42^ and Wallace and Happé^22^, but visual in a study by Gil *et al*.^25^ and our study) or whether the intervals were unfilled (defined by two events, as in a study by Allman^32^ and our study) or filled (as in a study by Gil *et al*.^25^) are worth exploring in the future. The general heterogeneity of autistic population, evidenced by inconsistent findings across a range of domains^18^,^43^ should also be considered to account for variable findings.

Notwithstanding, for the goals of this study, the most important result is that the autistic children do not use prior knowledge to reduce errors in an optimal manner, as revealed by a series of computational simulations (Figs 5, 7 and 8). This result was based on the comparison of autistic children to the youngest group of typical children. Six-year-old typically developing children also have low temporal resolution, comparable to that of the older autistic participants. However, they also have much stronger Bayesian priors, appropriate for their reduced temporal sensitivity.

These results provide further support for the idea that autistic perception is associated with reduced use of predictive information, as encoded in internal models for the recent history of the sensory input^26^. Although there is evidence of regression to the mean in the data of autistic children, the amount of regression is less than is required for optimal perception, given the reduced sensitivity for temporal discrimination. In other words, the degree of regression is not enough to compensate for their poor temporal resolution. Our findings reinforce other studies suggesting that internal priors are underweighted or less used in autistic children than in typical children (adaptive coding in face perception^44^,^45^ and number perception^46^, though see another study on perceptual causality^47^). Our approach also presents correspondences to alternative accounts of autistic perception suggesting inflexible weighting of cues by their estimated uncertainty^31^, although to examine explicitly this hypothesis one should vary the range of the intervals in two sessions of the time reproduction task (which was not the case in our paradigm).

Brock^27^ argued that, within a Bayesian model of perceptual inference, an asymmetric integration of prior knowledge with sensory estimates in autism could arise not only from attenuated prior knowledge (as posited by Pellicano and Burr^48^) but also from sensory estimates that are less noisy (akin to accounts positing a ‘bottom-up’ perceptual deficit in autism^27^). Our study does not support the latter possibility. Our data suggest that temporal estimates are indeed noisier in autism; however prior knowledge representations are also atypical as predicted by Pellicano and Burr^26^,^48^.

The reduced levels of central tendency (given their temporal resolution) might suggest autistic children have difficulties in restraining sensory noise, consistent with accounts of increased sensory noise in autism^18^). Autistic children do not use internal representations of an average stimulus as efficiently as typical children to moderate their noisy temporal resolution.

Our raw data suggest a consistent underestimation of almost all durations in all child groups, which did not occur in the earlier adult studies^8^,^9^. It is possible that this discrepancy results from methodological differences between our adapted child-friendly paradigm and the original paradigms^8^,^9^ (e.g., number of trials, range of tested stimuli). Nevertheless, we would argue that there are two different and separable effects in our time reproduction data: a consistent underestimation, and a compression of timescale. The underestimation biases did not affect the regression. As these biases were not the primary goal of the study, we did not investigate their origin, although they seem to decrease with age. However, a fuller account of time interval reproduction in typical and autistic children would need to address this issue in further detail.

Our data also present weaker context-dependency effects than the adult studies^8^,^9^ and the study of Sciutti *et al*.^7^ in the spatial domain. This may be due to various methodological differences, including a smaller number of trials, introduced with a child-appropriate task, which might have resulted in less statistical power in detecting context dependency in our paradigm.

Our model assumes the same levels of motor noise in typical and autistic children, although it is likely that the levels are elevated in autistic participants^49^. A fully defined model would need to constrain motor error. However, since increases in motor error influences the error of variability rather than regression and bias, our conclusions do not depend on the precise estimate of noise and are nevertheless valid if elevated levels of noise are implemented. Our model also assumes that prior knowledge representations are stationary during each session of the time interval reproduction task. This is a simplifying assumption of the model. A fully defined model would need to capture the learning or the progressive use of a prior during a session evidenced by the increase of regression indices from the first to the second half of a session in our split-half analysis. Finally, our approach does not distinguish between the nature of the atypicality in autistic children – that is, whether the atypicalities involve the learning of the prior or the use of priors, or indeed if the same model applies to different groups (either developmental or autistic vs. typical).

Notwithstanding, our approach situates the anecdotally reported timing difficulties of autistic individuals within a more general framework of Bayesian perceptual inference, rather than suggesting a specific timing deficit in autism^32^,^33^. Crucially, this approach applies to magnitude estimation in other domains. For example, it predicts a similar pattern of results (less regression for autistic children given their spatial resolution) in a spatial interval reproduction paradigm like that of Sciutti *et al*.^7^. Furthermore, our approach unifies magnitude estimation difficulties in autism to a broader range of sensory symptoms in the conditions, such as hypersensitivities and hyposensitivities (e.g., to sounds or lights). All these problems, which can be very disturbing and stressful to autistic people, stem from inflexible perceptual processing, in particular from problems in effectively adapting to and calibrating against observed sensory evidence.

## Methods

### Participants

Participants’ demographics are shown in Supplementary Table S1.

#### Typically developing children

Seventy-eight typically developing children and adults participated in this study, including twelve 6–7 year-old children (*M* = 7 years; 0 months, range: 6; 0–7; 10, 9 females), nineteen 8–9 year-olds (*M* = 9 years; 0 months, range: 8; 1–9; 10, 7 females), eighteen 10–11 year-olds (*M* = 11 years; 1 months, range: 10; 2–11; 11, 11 females), fifteen 12–14 year-olds (*M* = 12 years; 9 months, range: 12; 0–14; 1, 10 females) and fourteen adults (*M* = 25 years; 11 months, range: 22; 0–32; 0, 10 females).

#### Autistic children

Twenty-three autistic children (*M* = 12 years; 4 months, range: 7; 9–14; 8, 6 females) were recruited from schools in Greater London and community contacts. They had previously been diagnosed with autism or Asperger syndrome by independent clinicians according to the International Classification of Diseases criteria (ICD-10)^50^ or the criteria of the Diagnostic and Statistical Manual of Mental Disorders (DSM-IV)^51^. All autistic children obtained IQ scores of 70 or greater on the Wechsler Abbreviated Scales of Intelligence - 2nd edition (WASI-II)^52^ (*M* = 100.30, *SD* = 15.72, range = 7–128) and were thus considered cognitively able. Autistic children were also administered the Autism Diagnostic Observation Schedule – Generic (ADOS-G)^53^, while the parents/carers of autistic children completed parental report versions of the Social Communication Questionnaire^54^. Children were included in data analysis if they had an independent clinical diagnosis of autism and scored above threshold for an autism spectrum disorder in either the ADOS-G or the SCQ^55^ (ADOS-G cut-off score = 7^53^; SCQ cut-off score = 15^54^).

#### Typically developing comparison children

A subset of the typically developing children (n = 23; *M* = 11 years; 8 months, range: 7; 8–13; 10, 10 females) were also matched to autistic children in terms of chronological age, *t*(44) = 1.40, *p* = 0.17, non-verbal ability, *t*(44) = 0.19, *p* = 0.85, and verbal ability, *t*(44) = 0.96, *p* = 0.34, as measured by the WASI-II^52^.

#### Exclusions

An additional eight typically developing children (five 6–7 year-olds, two 8–9 year-olds, two 10–11 year-olds, and one 12–13 year-old) and an additional six autistic children were also tested but excluded from all analysis due to poor performance in the time discrimination task (Weber Fraction out of the range of [0, 1]).

### Apparatus and Stimuli

Stimuli were presented on the 15.6″ LCD screen (refresh rate: 40 Hz; pixel resolution: 1366 × 768) of a Dell Precision M4700 laptop using MATLAB (The Mathworks Inc., Natick, Massachusetts, USA) and routines of the Psychophysics Toolbox software^56^,^57^. The screen was black with a fixation pattern, consisting of four dots (0.5°) arranged at the vertices of a 5°-edge centrally-aligned square, presented on it throughout each task.

In both tasks, stimuli were green discs with raised cosine edges and a diameter of 5° of visual angle when presented at a 57 cm distance. The discs were presented on a black background for 200 ms, i.e., 8 frames in the refresh rate used. They were centred 5° above centre-screen, exactly on centre-screen (0°), and 5° below centre-screen depending on whether they corresponded to the first, second and third stimuli in a trial.

### Procedure

**Figure 1:**
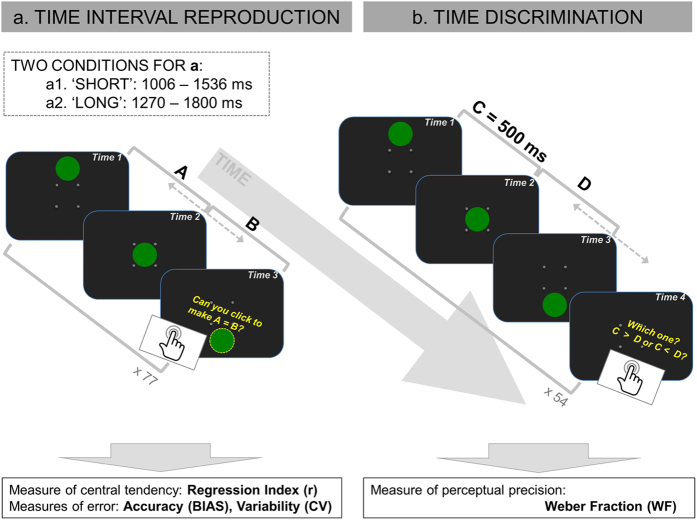
Graphical illustration of the study design.

**Figure 2:**
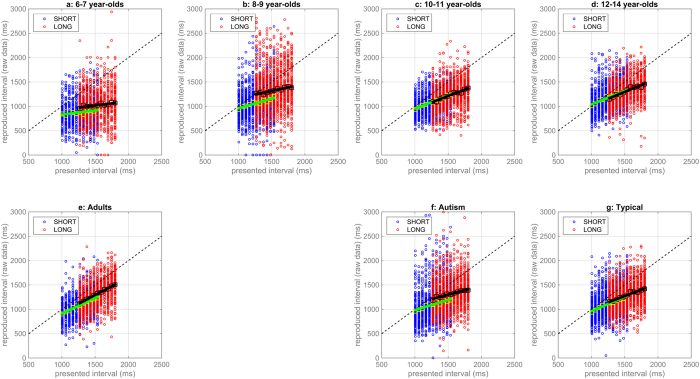
Responses in the two sessions of the time interval reproduction task for different participant groups (Panels (**a**–**d**) typical children; (**e**) adults; (**f**) autistic children; (**g**) typical comparison children). Small dots correspond to individual participants’ responses for short (blue) and long (red) intervals; squares correspond to group averages. Green and black continuous lines represent linear fits of the data from the short and long interval condition, respectively. The dashed black lines are the equality line, i.e., veridical performance.

**Figure 3:**
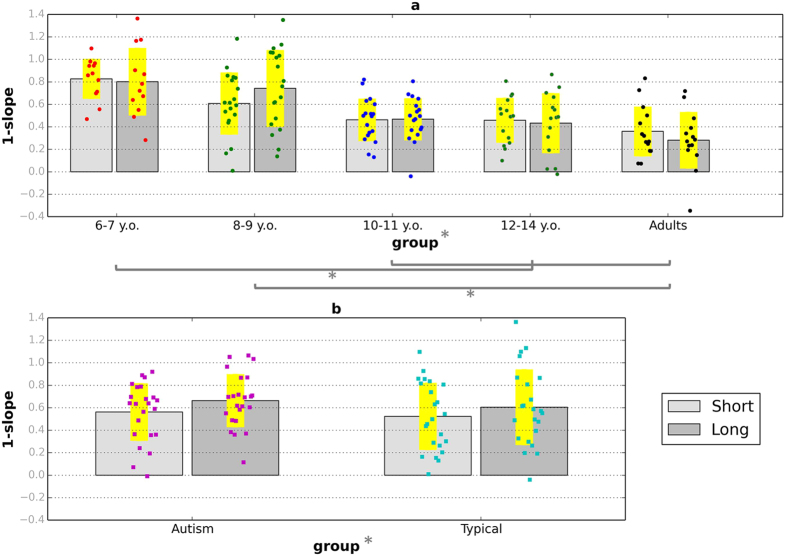
Regression indices for the short and long interval conditions of the time interval reproduction task. (**a**) Typically developing children and adults. (**b**) Autistic children and typically developing comparison children. Coloured dots show individual data and yellow bands represent ±1 SD. Stars indicate significant differences (*p* < 0.05).

**Figure 4:**
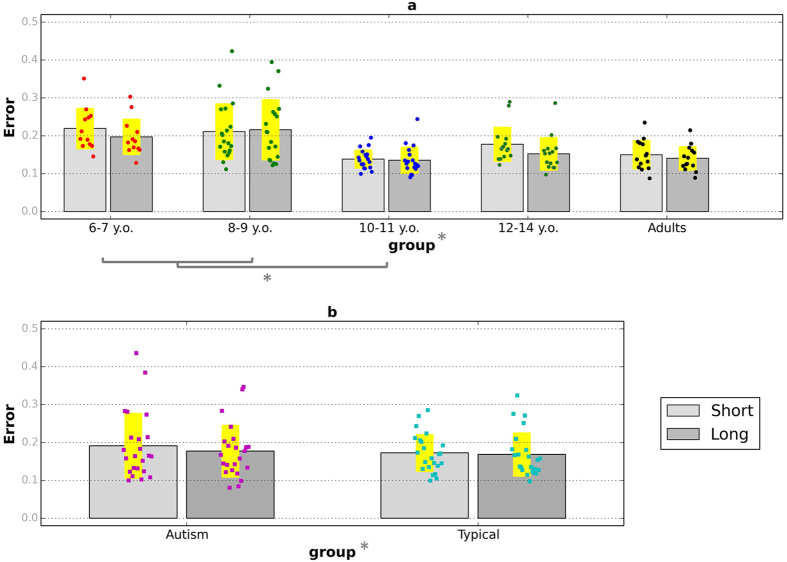
Total error for the short and long interval conditions of the time interval reproduction task. (**a**) The four age groups of typically developing children and adults; (**b**) autistic children and typically developing comparison children. Coloured dots show individual data and yellow bands represent ±1 SD. Stars indicate significant differences (*p* < 0.05).

**Figure 5:**
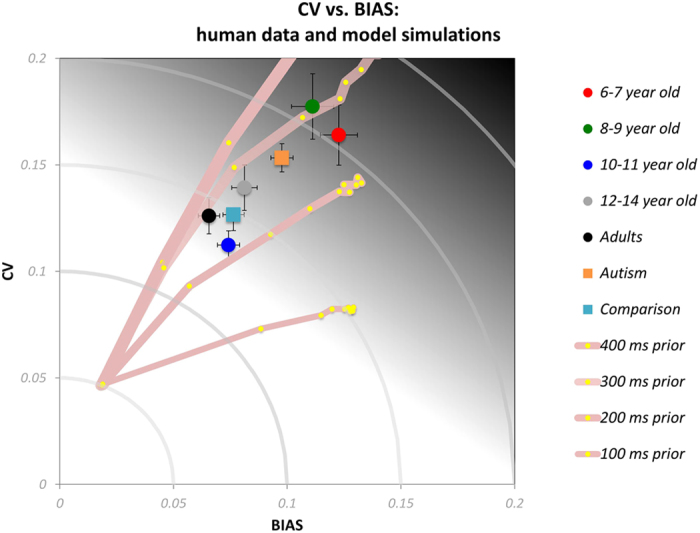
Partitioning of the total error in the time interval reproduction task: Coefficient of variation of the reproduction times (CV, Equation 9 in Supplementary Method) plotted against bias (BIAS, Equation 8 in Supplementary Method) for different participant groups. Circles correspond to typically developing children and adults (red: 6–7 year-olds, green: 8–9 year-olds, blue: 10–11 year-olds, grey: 12–14 year-olds, black: adults); squares to autistic children (orange) and the comparison typically developing children (light blue). Error bars represent ±1 SEM. The continuous grey lines correspond to predictions of the computational model for different value for different prior widths (400, 300, 200, and 100 ms) and Weber fractions in the range 0.01–1.00.

**Figure 6:**
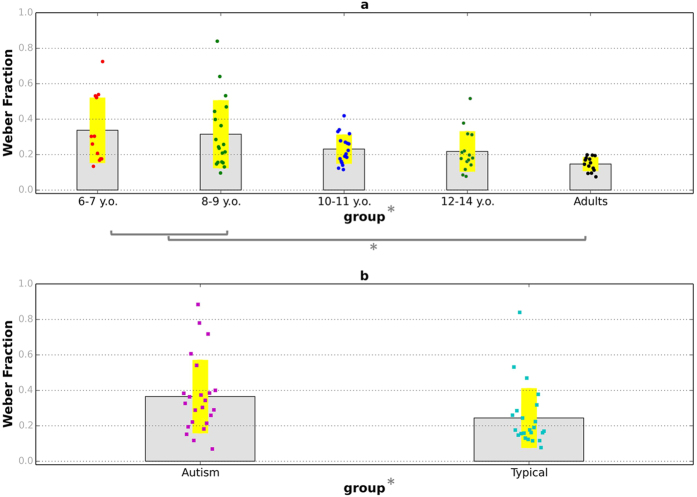
Precision in the time discrimination task. (**a**) Typically developing children and adults; (**b**) autistic children and typically developing comparison children. Coloured dots show individual data and yellow bands represent ±1 SD. Stars indicate significant differences (*p* < 0.05).

**Figure 7:**
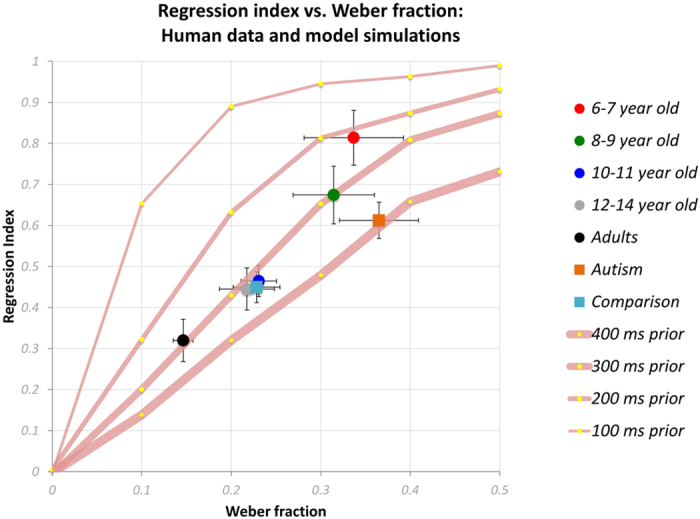
Regression index in time interval reproduction task plotted against Weber fraction in time discrimination. Circles correspond to typically developing children and adults (red: 6–7 year-olds, green: 8–9 year-olds, blue: 10–11 year-olds, grey: 12–14 year-olds, black: adults); squares to autistic children (orange) and the control group of typically developing children (light blue). Error bars represent ±1 SEM. The continuous lines correspond to predictions of the computational model considering a given value for the width of the prior (400, 300, 200, or 100 ms) and Weber fractions in the range 0.01–1.00.

**Figure 8:**
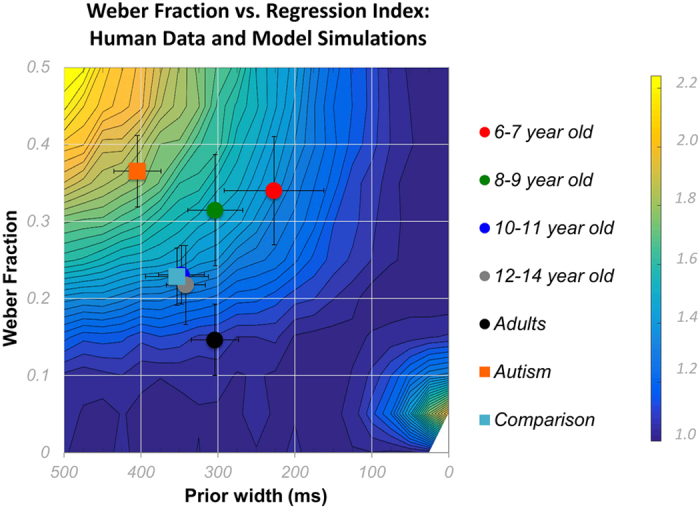
Error landscape, showing the relative RMS error for different Weber fractions (WF) and different prior widths (SP). Circles correspond to average data from typically developing children and adults (red: 6–7 year-olds, green: 8–9 year-olds, blue: 10–11 year-olds, grey: 12–14 year-olds, yellow: adults) and squares to average data from autistic children (orange) and typically developing comparison children (light blue). Human data are superimposed on the error surface with the estimates for prior widths of different groups derived from Equation 4.

Figure 1 provides a graphical illustration of the two tasks.

#### Time Interval Reproduction task

This task was administered as a “Ready, Set, Go!” paradigm and within the context of a cover story about Marco, a 3D animated character. The task comprised two sessions, one testing the reproduction of 11 ‘short’ durations, in the range 1006–1536 ms, and the other testing 11 ‘long’ durations, from 1270–1800 ms. Children completed seven trials per duration, yielding 77 testing trials per participant per session, separated into four blocks. For additional details on the administration of this task see Supplementary Method available.

#### Time Discrimination task

The discrimination task was administered within the same cover story as the time interval reproduction task. It comprised 54 test trials, divided into three blocks. In each test trial, a reference interval of 500 ms (equal to the range of tested intervals in the two conditions of the time interval reproduction task) was followed by a comparison interval. Two QUEST^58^ functions, starting with initial comparison intervals of 1200 ms and 200 ms, ran interleaved for 27 trials each to estimate the participant’s temporal resolution. For additional details on this task see Supplementary Method available.

#### General procedure

The study was conducted in accordance to the principles laid down in the Declaration of Helsinki. Ethics approval was granted by the UCL Institute of Education Research Ethics Committee and parents of all children gave informed consent prior to participation. Adults also gave their informed consent. Children were either tested in school, at the University or at the family home. All adults were tested at the University. The order of testing on the two conditions (short, long) of the interval reproduction task was counterbalanced across participants. Participants completed the discrimination task after one of the conditions of the reproduction task. Tests of intellectual functioning (WASI-II^52^) and autistic symptomatology (ADOS-G^53^) were administered in separate sessions.

### Analysis of empirical data

We used data from the time interval reproduction task to calculate regression indices (measurements of central tendency) for each session, as well as total error, which we divided to error or accuracy and error of reliability^8^,^9^. We used data from the time discrimination task to measure a Weber fraction for each participant, as a measure of his/her temporal resolution (see Supplementary Method available).

### Computational modelling

Modelling participants’ performance in the time interval reproduction was based on the modelling procedures of the previous studies^7^,^8^. We assumed that both the prior and the likelihood functions are normal distributions with means *μ*_*P*_ and *μ*_*L*_ and standard deviations (widths) *σ*_*P*_ and *σ*_*L*_, correspondingly. In a given session, the prior function is centred on the average stimulus duration 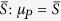. The likelihood function for stimulus *S*_*i*_ is centred on a noisy measurement of the stimulus, *t*_*i*_ ms away from the average stimulus: 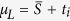. Applying Bayes’ rule, the posterior distribution (reproduced times) is a normal distribution with a mean: *μ*_*R*_:


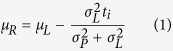


and a standard deviation *σ*_*R*_:


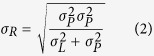


We also assumed added motor noise with standard deviation


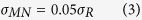


i.e. at a 5% level (as in Cicchini *et al*.^8^).

#### Modelling performance in time interval reproduction under the assumption of different prior widths

In a first set of computational simulations, we simulated the performance of participants who completed the two sessions of the time interval reproduction task employing prior knowledge distributions of different widths (assumed to be constant during the progression of each session task). In particular, we considered prior widths of 400 ms, 300 ms, 200 ms and 100 ms – that is, corresponding to 80%, 60%, 40% and 20% of the reference interval of the time discrimination task. We combined these prior widths with Weber fraction values from the range 0 to 1 (with an increment of 0.1) considering 100 repetitions for each combination.

For each prior width/Weber fraction combination, we calculated the values predicted by the model for the following measures: regression index *r* (averaged across the two sessions) and the two components of error, *BIAS* and *CV* (averaged across sessions and across stimuli). We compared the empirical data from different participant groups to the predictions of the computational model by superimposing the human and the computational modelling data on two diagrams: one plotting regression indices against Weber Fractions and one plotting *CV* (response variability) against *BIAS* (response accuracy).

#### Assessing relative error

In a complementary set of simulations, we used the model to generate a so-called relative error landscape. This landscape, which assigned a relative error value to different prior width/Weber fraction combinations, indicated the extent to which different prior widths minimised overall error for a given Weber fraction value. For a given Weber fraction value, the relative error was the total reproduction error normalised across the range of different priors.

We assessed the extent to which the different groups performed optimally in the time interval reproduction by superimposing the human data on the relative error landscape. The superimposition of empirical data on the simulated error landscape took into account the averaged Weber fraction values across groups, as obtained from the time discrimination task, and estimates of the width of the prior knowledge distribution of different groups. The estimates for the standard deviation of prior knowledge were a function of participants’ regression indices (*r*) and the Weber fractions (*WF*):


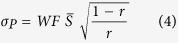


where 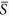 is the mean stimulus interval. Equation 4 was derived from the following formula for the slope of the fitted curve to the reproduced time intervals in a group:


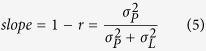


where *σ*_*L*_, the width of the likelihood function, is a function of Weber fraction (*WF*) and the mean stimulus in a session 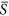:


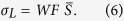


The standard deviations of the priors and the likelihood were calculated from Monte Carlo simulations with 10,000 repetitions.

We note that an intuitive interpretation of Equation 5 is that when temporal discrimination is highly precise 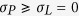) then the slope of the fitted curve is 1, corresponding to veridical reproduction of time intervals. When, however, temporal resolution is poor 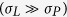, the slope is 0, corresponding to full regression.

We also note that the estimates of the standard deviation of the prior and the likelihood (Equations 4 and 6) are based on the assumption that the WF measured experimentally in the time discrimination task may be transferred to the time reproduction task. This approach has been successful in previous studies on interval reproduction^7^,^8^,^9^, as well as in multistable perception^59^,^60^.

## Additional Information

**How to cite this article**: Karaminis, T. *et al*. Central tendency effects in time interval reproduction in autism. *Sci. Rep.* **6**, 28570; doi: 10.1038/srep28570 (2016).

## Supplementary Material

Supplementary Information
