# Supplementary material for: Central tendency effects in time interval reproduction in autism
Source: Sci Rep. 2016 Jun 28;6:28570. doi: 10.1038/srep28570 (PMC4923867; doi:10.1038/srep28570)

Supplementary materials for "Central tendency effects in time interval reproduction in autism"

Themelis Karaminis[1, 2, *], Guido Marco Cicchini[3], Louise Neil[1], Giulia Cappagli[1, 4], David Aagten-Murphy[5], David Burr[3, 6], and Elizabeth Pellicano[1, 6]

[1]Centre for Research in Autism and Education (CRAE), Department of Psychology and Human Development, UCL Institute of Education, University College London, London, UK

[2]School of Psychology, Plymouth University, Plymouth, UK

[3]Institute of Neuroscience, National Research Council (CNR), Pisa, Italy

[4]Istituto Italiano di Tecnologia, Genova, Italy

[5]Department of Psychology, Ludwig-Maximilians-Universität München, Münich,Germany

[6]School of Psychology, University of Western Australia, Perth, Australia

[*]t.karaminis@ioe.ac.uk, themkar@gmail.com

Supplementary Method

Supplementary Table S1. Participant demographics.

Group	6 – 7 year-olds	8 – 9 year-olds	10 – 11 year-olds	12 – 13 year-olds	typical adults	autistic children	comparison children
**n**	12	19	18	15	14	23	23
male : female	*3 : 9*	*12 : 7*	*7 : 11*	*5 : 10*	*4 : 10*	*17 : 7*	*13 : 10*
**Age** (years; months)							
Mean	7; 0	9; 0	11; 1	12; 9	25; 11	12; 4	11; 8
SD	*0; 7*	*0; 7*	*0; 7*	*0; 6*	*3; 9*	*1; 10*	*1; 7*
Range	6; 0 – 7; 10	8; 1 – 9; 10	10; 2 – 11; 11	12; 0 – 13; 11	22; 0 – 32; 0	7; 9 – 14; 8	7; 8 – 13; 10
**V-IQ**							
Mean	104.67	108.05	107.28	98.13		103.30	101.09
SD	*10.06*	*8.47*	*12.22*	*10.78*		*15.08*	*11.23*
Range	90 – 124	98 – 133	90 – 130	79 – 120		75 – 128	79 – 126
**P-IQ**							
Mean	103.17	101.32	102.11	100.47		103.30	104.04
SD	*12.63*	*14.08*	*12.98*	*10.91*		*15.08*	*11.16*
Range	85 – 130	74 – 128	75 – 123	80 – 117		75 – 128	80 – 123
**FS-IQ**							
Mean	104.33	105.37	105.28	99.13		100.30	102.78
SD	*11.36*	*10.00*	*11.33*	*10.23*		*15.72*	*11.26*
Range	90 – 123	88 – 120	86 – 125	84 – 122		70 – 128	84 – 125
**ADOS**						(n = 20)	
Mean						9.35	
SD						*2.89*	
Range						6-16	
**SCQ**	(n = 7)	(n = 18)	(n = 12)	(n = 8)		(n = 21)	(n = 14)
Mean	3.00	4.39	2.92	1.50		26.71	2.07
SD	*2.44*	*4.53*	*1.88*	*2.00*		*9.45*	*2.06*
Range	0 – 7	0 – 14	0 – 6	0 – 6		10 – 46	0 – 7

V-IQ, P-IQ, FS-IQ: Verbal, Performance and Full-Scale IQ scores derived from the Wechsler Abbreviated Scales of Intelligence 2nd edition (WASI-II)[52]; ADOS = Autism Diagnostic Observation Schedule [53]; SCQ = Social Communication Questionnaire [54]. Higher scores on the ADOS and SCQ reflect greater degrees of autistic symptomatology.

Additional details on the two tasks

Time Interval Reproduction task

This task was administered as a "Ready, Set, Go!" paradigm administered within the context of a cover story about Marco, a 3D animated character who worked at the local discotheque and whose job it was to set up the timing of the disco lights. The disco lights were flashes (green discs) that appeared successively on the screen, with certain time distances between them. Each trial began with children seeing two flashes (Ready, Set), the first centred 5° above centre-screen and the second centred exactly on centre-screen (0°). Children were asked to click the mouse to tell Marco when a third flash should be produced (Go!). They were instructed to keep the same distance in time

between the first and the second flash as between the second flash and when clicking the mouse.

The session started with children watching a short animation about Marco and were explained the task. They were then shown two example trials, which allowed the experimenter to illustrate the task and explain the game in further detail. Next, they performed four practice trials, the first two including visual feedback where the child saw a flash, centred 5° below centre-screen, upon clicking the mouse. No feedback was given for the final two practice trials to prevent the child adopting strategies based on the perception of three consecutive flashes rather than the interval between the first two flashes.

Practice trials were followed by two test sessions, one comprising short intervals in the range 1006 - 1536 ms and the other long intervals from 1270 - 1800 ms. The order of the two sessions was counterbalanced across participants and were administered after a 2 - 3 hour break between them. Each session focussed on the reproduction of eleven durations from the corresponding range, six of which were common to both sessions. Children completed seven trials per duration, yielding 77 testing trials per participant per session, separated into four blocks. The whole task lasted 35 - 40 mins.

Time Discrimination task

In this task, children were instructed to help Marco with another activity related to the timing of lights. On each trial, they saw three flashes that appeared on screen with different time distances between them (in the range 200 ms 1200 ms). The first flash appeared 5°above centre-screen, the second exactly in centre-screen (0°) and the third 5°below centre-screen. Children were instructed to judge whether the middle flash was closer in time to the first flash or the last flash and to make an appropriate key press (labelled as 1 and 3) to indicate their judgement.

The discrimination task was structured in a similar way to the time interval reproduction task. It began with an introductory phase, which allowed the experimenter to explain the task, followed by four practice trials. Practice trials started with an easy-to-identify difference (first interval : second interval = 2.35, based on piloting) and ended with a more challenging example (first interval : second interval = 1.6). Children received feedback regarding accuracy during the practice phase. This phase was repeated with children who were not successful in all practice trials (two 6 - 7 year-olds and two children with autism).

During the test phase of the discrimination task, a reference interval of 500 ms (equal to the range of tested intervals in the two conditions of the time interval reproduction task) was followed by a comparison interval. Two QUEST [58] functions, starting with initial comparison intervals of 1200 ms and 200 ms, ran interleaved for 27 trials each (each QUEST had a beta value of 3 and a lapse rate set to 0.01). The QUEST homed in on the point where the two intervals appeared equal; to ensure a good distribution of durations to estimate discrimination thresholds, a random jitter of SD = 60 ms was also added to the QUEST estimates [58]. Threshold estimates were taken as the standard deviation of the best-fitting cumulative Gaussian (see Cicchini et al.[8] for details). Participants performed 54 test trials and an additional 6 catch trials (non-QUEST trials, aiming to correct for automatic responses/guesses).

The trials were divided into three separate blocks (in the context of the cover story: sets of lights that should be measured).

Measurements and analysis

Each session of the interval reproduction task included 11 possible intervals repeated 7 times for a total of 77 trials. Each session therefore provided 77 measurements of reproduced intervals $R_{i,n}$, where i is an interval index, ranging from 1 to 11, and n is its repetition, ranging from 1 to 7. We took all measurements greater than 3000 ms as attention lapses and excluded these from analysis. We also considered all measurements exceeding 2.5 standard deviations above or below the mean reproduced interval in each session as outliers.

Following Cicchini et al.[8], we generated corrected reproduction times $R'_{i,n}$, by subtracting the average reproduction time across all trials in session $\bar{R}$ from each $R_{i,n}$ and adding average stimulus duration $\bar{S}$:

$$R'_{i,n} = R_{i,n} - \bar{R} + \bar{S} \tag{7}$$

Corrected reproduction times allowed us to rule out any idiosyncratic and systematic overestimation or underestimation of time intervals and therefore focus on central tendency per se. That is, once the systematic overestimation or underestimation is discarded, we were left with a non-unity slope, implying regression towards the mean.

Regression index

The main measurement characterising performance in a given session of the interval reproduction task was the regression index r. The regression index was the difference between the slopes of a line fitted to the corrected reproduction times $R'_{i,n}$ and the equity line. It ranged from 0, corresponding to veridical performance, to 1, corresponding to complete regression to the mean. Any systematic biases (over- or underestimations) did not affect the regression index. That is, regression indices would be the same if calculated using raw or corrected times.

Context dependency

An important signature of central tendency effects in the reproduction task is that the estimations of the five interval values that were common in the two distributions should depend on the temporal context in which they were presented, being longer in the long than in the short condition [7]. We assessed the extent to which a given participant presented such context dependency by measuring the difference between the means of the distributions of the reproduced intervals for stimuli common to the two conditions.

Error and error partitioning

Following Cicchini et al.[8] and Jazayeri and Shadlen[9], we took error in the estimation of interval i to consist of two parts, the $BIAS$ or error of accuracy, which corresponded to the systematic offset from the real value of the

interval due to central tendency and the coefficient of variation (CV) or error of reliability, which reflected the degree of variability around the mean. The $BIAS$ for a given stimulus duration i was expressed as the difference between the average reproduced value (corrected) and the stimulus duration, normalised by the average stimulus duration in the session:

$$BIAS_i = \frac{R'_i - S_i}{\bar{S}} \tag{8}$$

CV for interval i was given by the standard deviation of the normalised reproduced times, normalised by the mean stimulus duration in the session:

$$CV_i = \frac{\sqrt{\Sigma(R'_i - \bar{R}'_i)^2/N}}{\bar{S}} \tag{9}$$

The total error in the estimation of interval i was the root mean squared error of $BIAS_i$ and CV_i:

$$TE_i = \sqrt{BIAS_i^2 + CV_i^2} \tag{10}$$

We note that the use of corrected (instead of raw) reproduction times in Equations 8 and 9 resulted in smaller values of BIAS for interval i (excluding all systematic over- or under estimation) but did not change CV, reflecting the variability or scatter of responses, which was the same in raw and corrected times.

We also considered the ratio $CV/BIAS$ to characterise the performance of participants in the time interval reproduction task based on the relative proportion of the two components of error. Higher values of this ratio would suggest that participants under- or over-estimated time intervals, but were generally accurate (showing little central tendency) in the time interval reproduction task; lower values would suggest participants with high levels of central tendency, but little scatter in their responses.

Weber fraction

From the temporal discrimination task, we calculated a Weber fraction values for each participant after fitting a cumulative Gaussian curve to participants' responses using a maximum likelihood method. The Weber fraction was given by the slope (variance) of the psychometric function divided by the offset (mean).

Supplementary Figures

Supplementary Figure 1. Regression indices for first and the second half of the session (averaged across conditions). a: typically developing children and adults. b: autistic children and typically developing comparison children. Coloured dots show individual data and yellow bands represent ±1 SD. Stars indicate significant differences ($p < 0.05$).

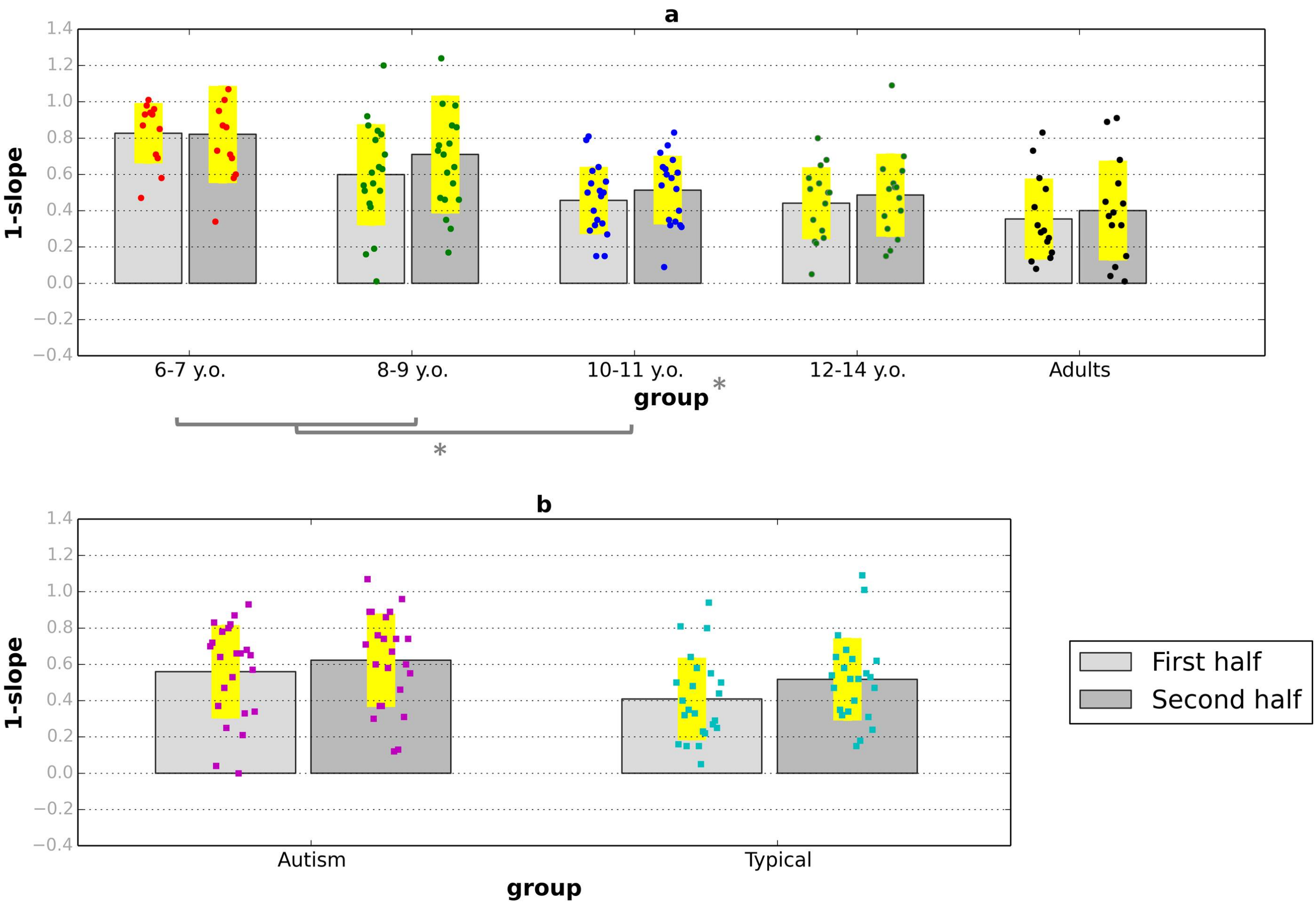

Supplementary Figure 2. Partitioning of the total error in the time interval reproduction task: Coefficient of variation of the reproduction times (CV, Equation 9) plotted against bias (BIAS, Equation 8) for different participant groups and for the two conditions, Short and Long. Circles correspond to typically developing children and adults (red: 6 - 7 year-olds, green: 8 - 9 year-olds, blue: 10 - 11 year-olds, grey: 12 - 14 year-olds, black: adults); squares to autistic children (orange) and the comparison typically developing children (light blue). Error bars represent ±1 SEM. The continuous grey lines correspond to predictions of the computational model for different value for different prior widths (400, 300, 200, and 100 ms) and Weber fractions in the range 0.01 - 1.00.

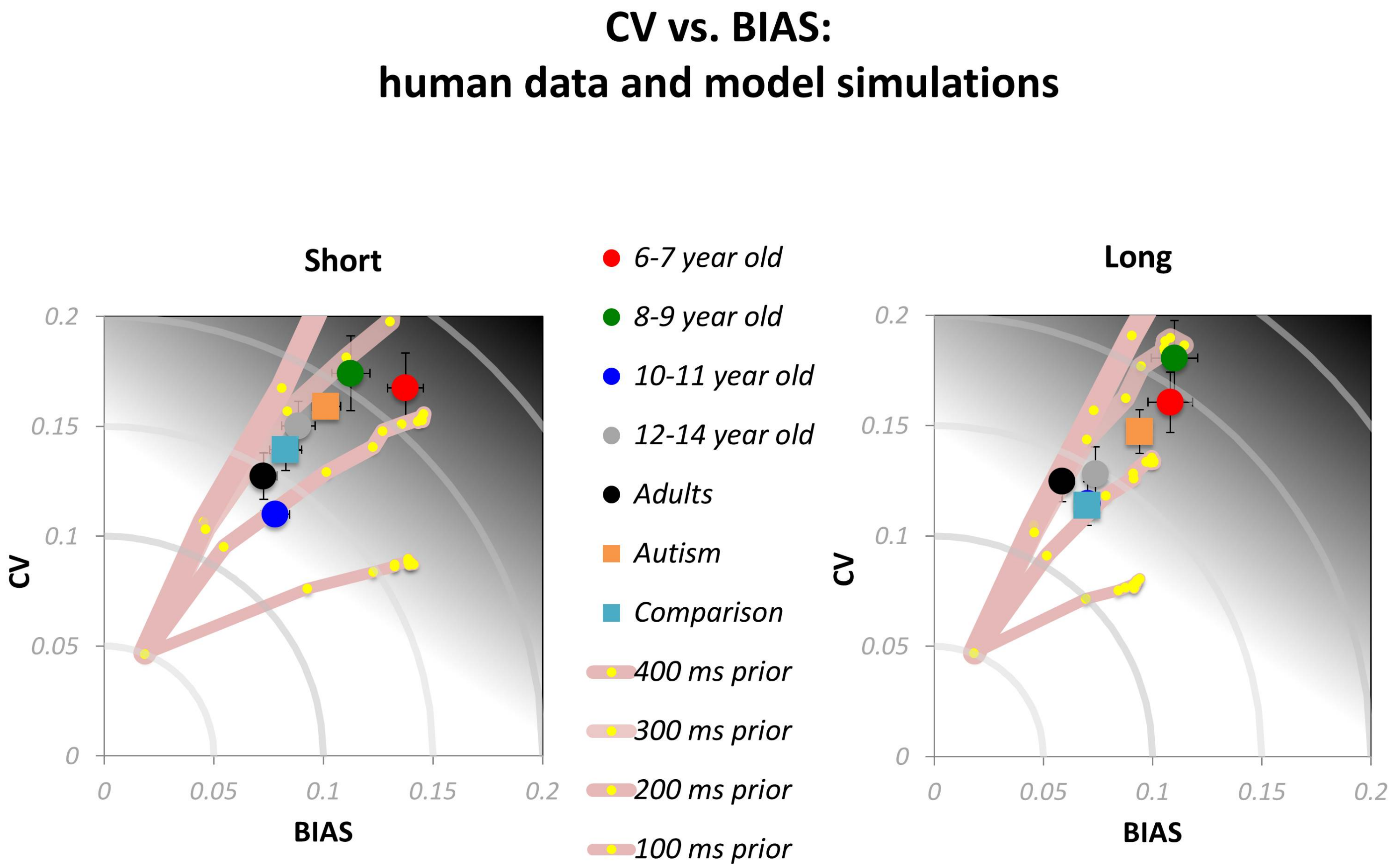

Supplementary Figure 3. Regression indices in time interval reproduction task plotted against Weber fraction in time discrimination for the two conditions, Short and Long. Circles correspond to typically developing children and adults (red: 6 - 7 year-olds, green: 8 - 9 year-olds, blue: 10 - 11 year-olds, grey: 12 - 14 year-olds, black: adults); squares to autistic children (orange) and the control group of typically developing children (light blue). Error bars represent ±1 SEM. The continuous lines correspond to predictions of the computational model considering a given value for the width of the prior (400, 300, 200, or 100 ms) and Weber fractions in the range 0.01 - 1.00.

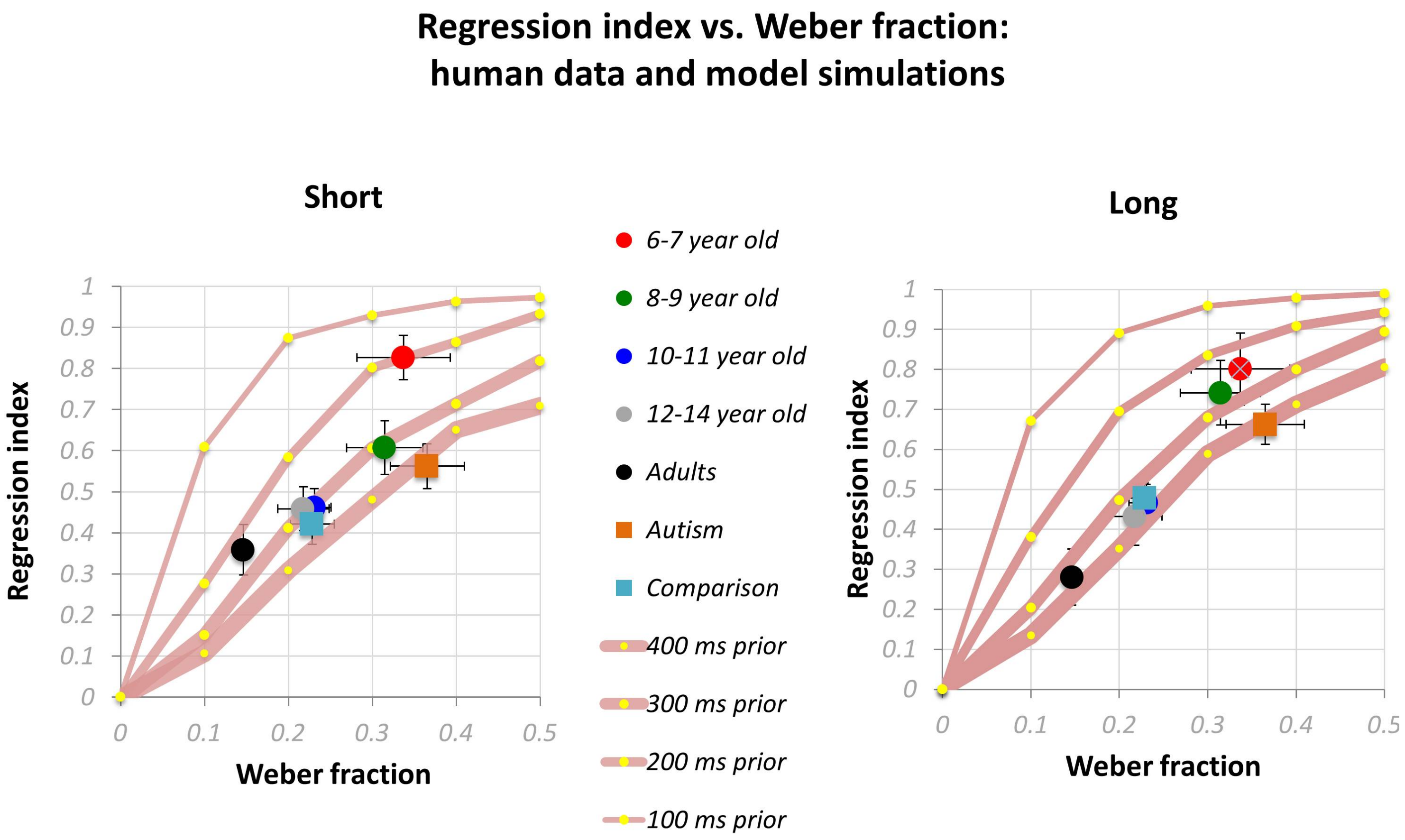

Supplementary Figure 4. Error landscape, showing the relative RMS error for different Weber fractions (WF) and different prior widths (SP), for the two conditions Short and Long. Circles correspond to average data from typically developing children and adults (red: 6 - 7 year-olds, green: 8 - 9 year-olds, blue: 10 - 11 year-olds, grey: 12 - 14 year-olds, yellow: adults) and squares to average data from autistic children (orange) and typically developing comparison children (light blue). Human data are superimposed on the error surface with the estimates for prior widths of different groups derived from Equation 4.

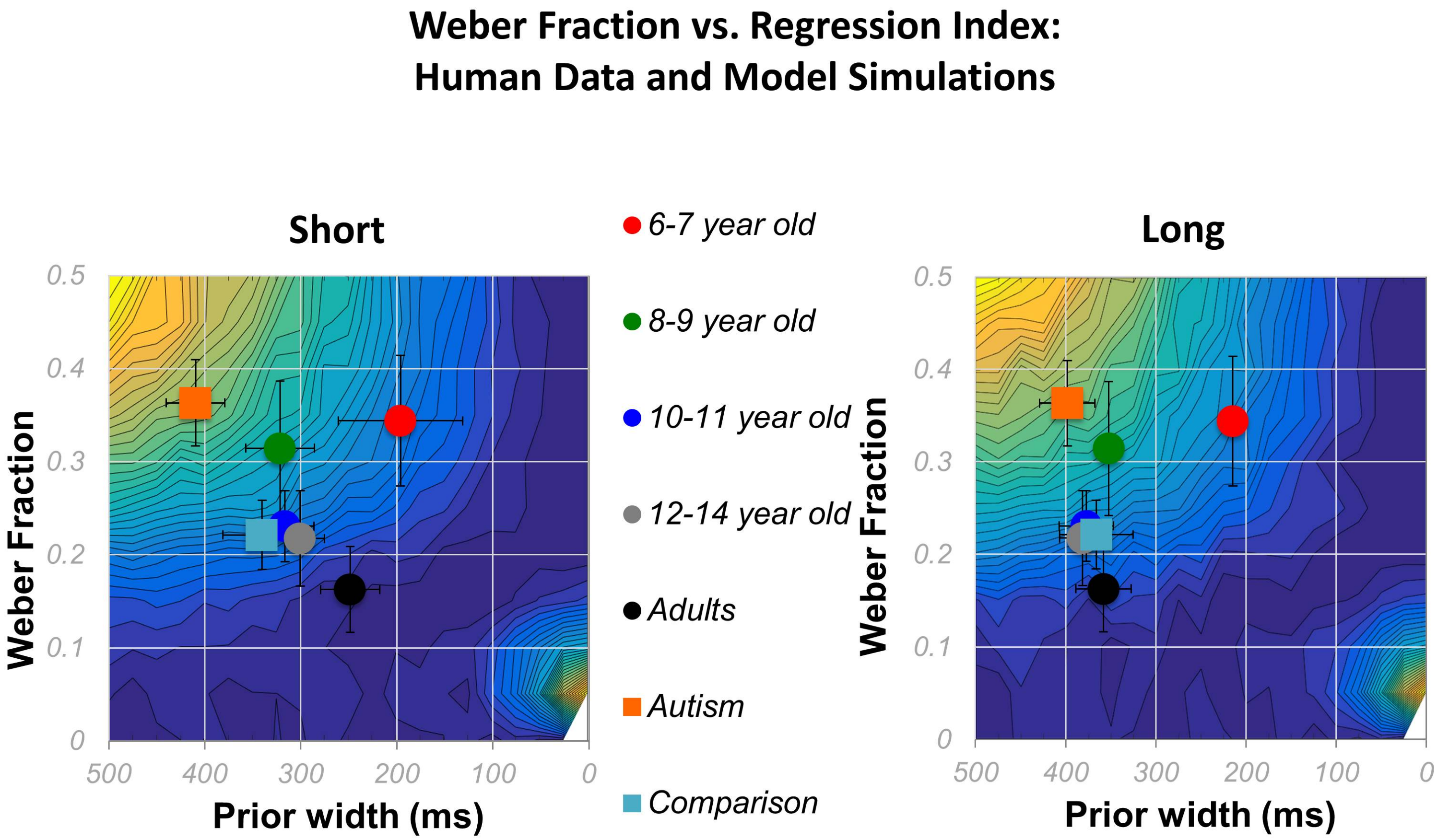

Supplement: Supplementary Information [file srep28570-s1.pdf]
